# Parallel processing of polarization and intensity information in fiddler crab vision

**DOI:** 10.1126/sciadv.aax3572

**Published:** 2019-08-21

**Authors:** Samuel P. Smithers, Nicholas W. Roberts, Martin J. How

**Affiliations:** School of Biological Sciences, University of Bristol, Bristol Life Sciences Building, 24 Tyndall Avenue, Bristol BS8 1TQ, UK.

## Abstract

Many crustaceans are sensitive to the polarization of light and use this information for object-based visually guided behaviors. For these tasks, it is unknown whether polarization and intensity information are integrated into a single-contrast channel, whereby polarization directly contributes to perceived intensity, or whether they are processed separately and in parallel. Using a novel type of visual display that allowed polarization and intensity properties of visual stimuli to be adjusted independently and simultaneously, we conducted behavioral experiments with fiddler crabs to test which of these two models of visual processing occurs. We found that, for a loom detection task, fiddler crabs process polarization and intensity information independently and in parallel. The crab’s response depended on whichever contrast was the most salient. By contributing independent measures of visual contrast, polarization and intensity provide a greater range of detectable contrast information for the receiver, increasing the chance of detecting a potential threat.

## Teaser

Fiddler crabs process intensity and polarization information independently and in parallel to enhance visual contrast.

## INTRODUCTION

Many animals, including insects, cephalopods, fish, and crustaceans, are sensitive to the polarization of light. Animals use this visual information for a variety of behavioral tasks such as navigation, communication, and habitat localization (*1*). Some animals can use the polarization of light for functional tasks that require the detection of a moving object, where polarization information is processed in a way that enhances visual contrast of the object against its background (*2*–*4*).

There are three known arrangements of polarization-sensitive photoreceptors that are able to provide contrast enhancement in image-forming vision (*5*). The most common of these photoreceptor arrangements, which has been converged upon by at least two evolutionary lineages (arthropods and cephalopods) and forms the focus of this study, is the dipolat system, a two-channel arrangement in which photoreceptors are oriented perpendicularly to each other. In this system, an intensity-independent measure of polarization contrast may be produced through opponent processing between these two polarization-sensitive channels (*6*, *7*). Dipolatic receptor arrangements have been found in the image-forming eyes of many animals including insects (*8*–*10*), cephalopods (*11*), and crustaceans (*12*, *13*).

**Fig. 1:**
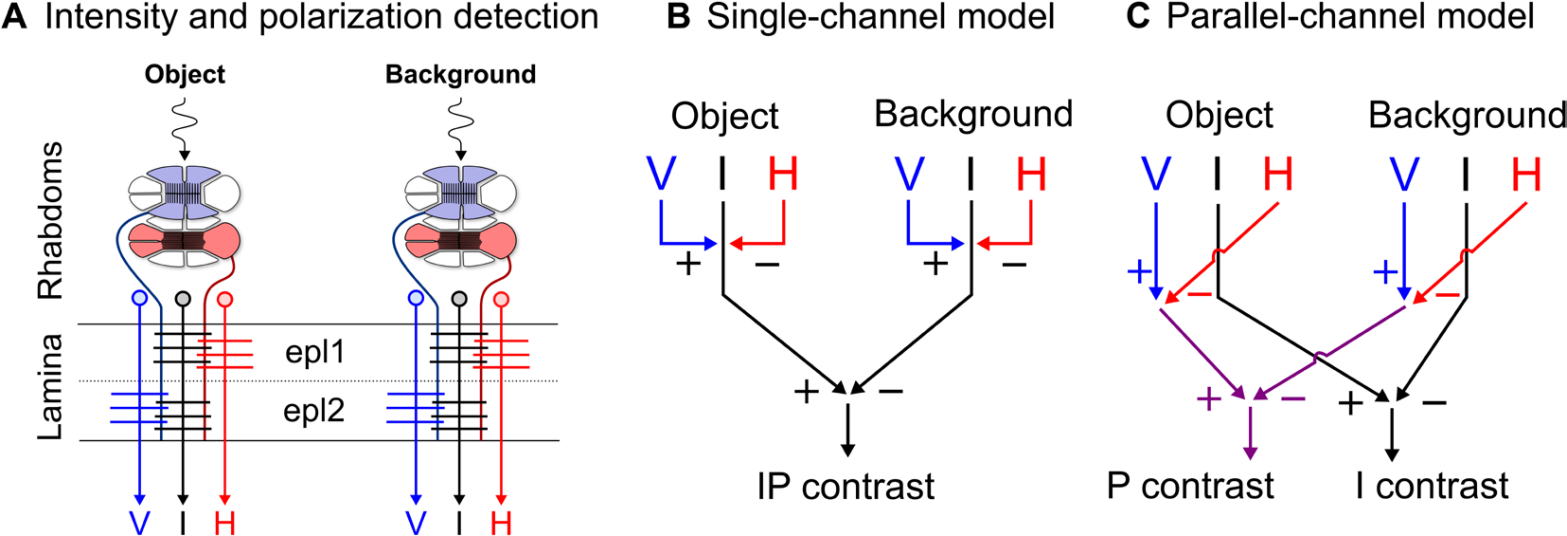
Hypothesized models of intensity and polarization channel integration in crustaceans. (**A**) Horizontally and vertically oriented receptor cells project to the epl1 and epl2 layers of the lamina, respectively, where they synapse with three types of descending neuron (monopolar cells M2 to M4), resulting in three channels of information per ommatidium: horizontal (H, M3) and vertical (V, M4) polarization, and intensity (I, M2) (*30*, *31*) [redrawn from (*7*)]. (**B**) Single-channel model demonstrating a fusion of V, H, and I into a single value [intensity-polarization (IP) contrast]. (**C**) Parallel-channel model in which the polarization (V and H) and intensity (I) channels combine separately into two parallel measures (P contrast and I contrast).

In crustaceans, such as fiddler crabs, this visual information is relayed from each of the perpendicularly oriented polarization-sensitive photoreceptors to the external plexiform layers (epl1 and epl2) of the lamina, where they synapse with three types of descending neurons: two that preserve the two channels of polarization information and one that sums their inputs to produce a polarization-independent brightness channel (Fig. 1A) (*14*, *15*). What is currently unknown, however, is how both polarization and intensity information are further processed, most likely within the medulla, to inform task-specific behaviors. Are these two forms of visual information combined together to provide the animal with a single, visual representation of overall contrast, or are they processed separately to provide independent and parallel measures of polarization and intensity contrast?

In dipolats, there is some evidence that could be consistent with animals integrating polarization and intensity information into a single-contrast channel in specific behavioral contexts. For instance, the crayfish *Procambarus clarkii* is known to respond to polarization contrasts almost identically to intensity contrasts (*16*, *17*). Moreover, larval stage *Anax imperator* (the emperor dragonfly) shows an increase in responsiveness to visual stimuli when viewed through a naturalistic horizontally polarized light field, which was equivalent to an increase in the intensity contrast of 8% (*4*). A possible explanation for equivalence in response to either intensity or polarization contrasts is that the two polarization channels [vertical (V) and horizontal (H)] combine with the intensity channel (I) (the three outputs from the lamina external plexiform layers; Fig. 1A) via excitatory and inhibitory synapses (single-channel model; Fig. 1B). However, such a single-contrast system would be subject to intensity/polarization cancellation points—situations where an animal would not be able to detect a visual contrast between an object and background despite differences in both intensity and polarization. In these cases, intensity and polarization channels would combine to cancel each other out.

Alternatively, polarization and intensity contrast within an image could be maintained and processed independently and in parallel, with these inputs being used in downstream processing circuits to mediate visually guided responses. This is somewhat analogous to our own intensity and color vision, in which each dimension contributes to its own measure of contrast in early visual processing [reviewed by Shapley (*18*)]. Here, we call this the parallel-channel model (Fig. 1C). While the previously measured behaviors could result from either of these models, it has never been explicitly tested which one underlies the connectivity of a dipolatic visual system for a specific behavioral task.

**Fig. 2:**
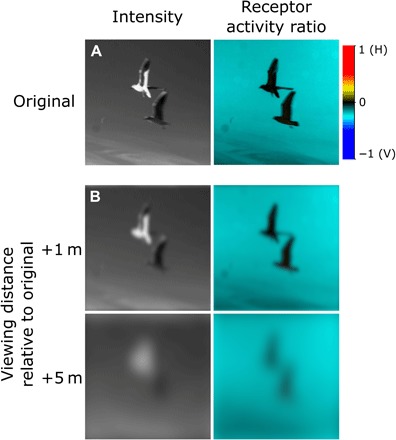
Intensity and polarization images of two black-headed gulls (*Chroicocephalus ridibundus*) viewed against a clear sky. (**A**) Original intensity and polarization images and (**B**) the same images showing the visual features that are resolvable by the crabs at increasing viewing distance based on the visual resolution of the region of the eye in *Gelasimus vomeris* [formerly *Uca vomeris* (*20*)] viewing approximately 15° to 20° above the horizon (*45*, *46*). The polarization information is presented as a receptor activity ratio, i.e., the relative opponent output of the horizontally (H = 1) and vertically (V = −1) oriented photoreceptor channels calculated using a visual model (*7*). Note how the intensity contrast of a predator can vary depending on the animal’s coloration and illumination, but the polarization contrast remains the same. The Supplementary Materials provide the details on the polarization video camera used to capture these images. Photo credit: Sam Smithers, University of Bristol.

There is a clear benefit for animals, and for crustaceans in particular, from using both intensity and polarization visual information independently. The mudflat environment in which fiddler crabs live is rich in polarization information, such as the polarization pattern of the sky and the predominantly horizontally polarized light reflected from damp areas of mudflat (*2*, *7*). Together, these different sources of polarized light form a polarized background against which approaching targets (typically unpolarized) are viewed, thus creating a valuable source of visual contrast in addition to intensity cues. For instance, the main predators of fiddler crabs are birds that walk or fly over the mudflats (*19*). As apparent in Fig. 2, different parts of an avian predator can appear darker or brighter than the background when viewed against a clear daytime sky, depending on their coloration, the illumination conditions, and the viewing direction. However, an avian predator, when viewed against a clear sky, will always be less polarized than the background, and thus, the opponent output of a dipolatic system (measured as receptor contrast in Fig. 2) remains constant even if the intensity contrast varies spatially and/or temporally. In these cases, polarization contrast can provide a more reliable signal than intensity; thus, using both polarization and intensity information is an advantage for crabs when detecting predators.

The aim of this study was to test whether a single- or parallel-channel processing model functions in an animal with dipolat polarization vision. To this end, we conducted a series of behavioral experiments with the fiddler crab *Afruca tangeri* [formerly *Uca tangeri* (*20*)], in which crabs were presented with a range of stimuli that differed in intensity and/or polarization.

## RESULTS

**Fig. 3:**
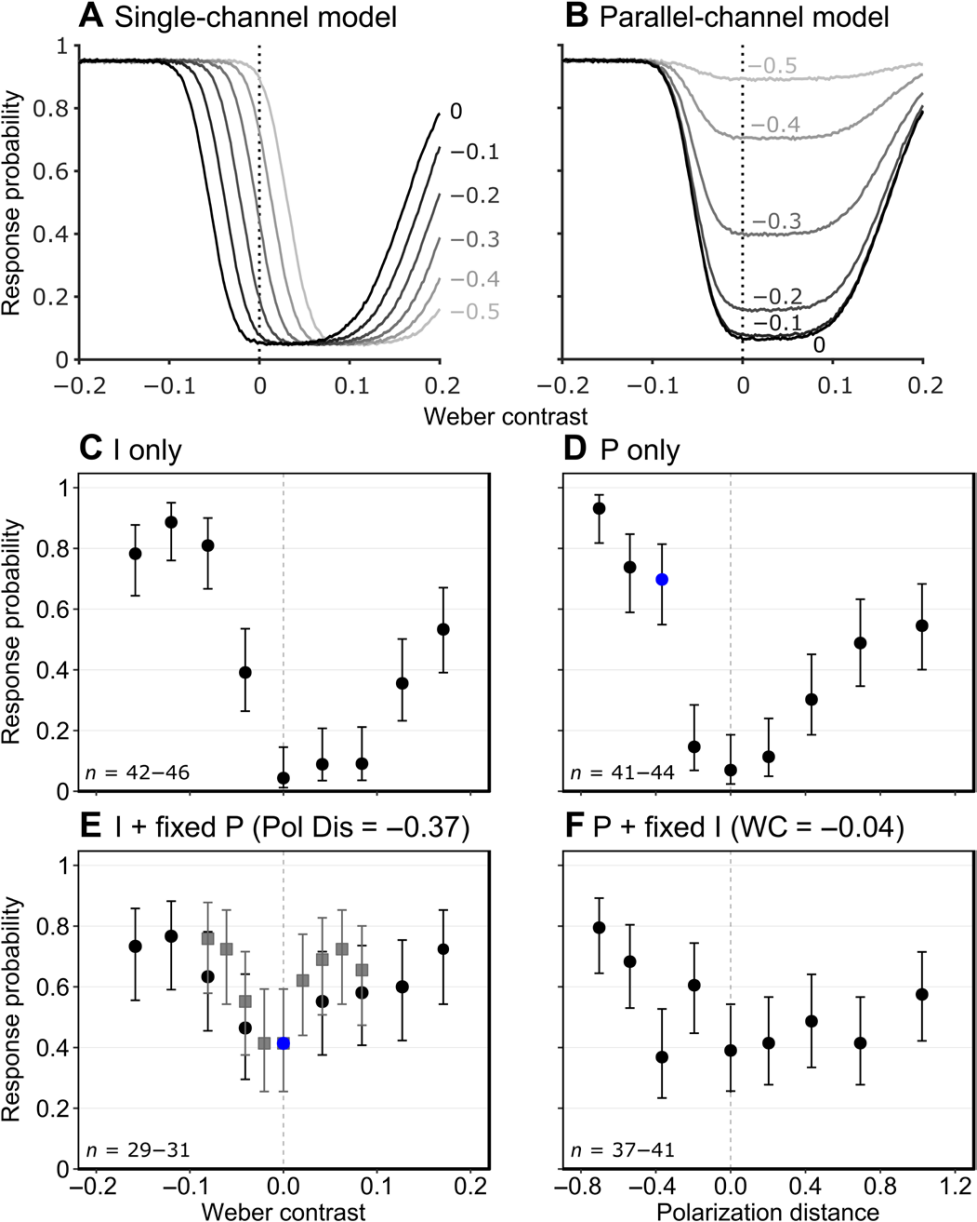
Predictions from the IP response models and results from behavioral experiments. The predicted response probabilities of a simulated crab population (*n* = 10,000) to a range of intensity contrasts, with the addition of a set of fixed polarization contrasts (polarization distance, 0 to −0.5; gray lines in increasing lightness) using (**A**) the single-channel model and (**B**) the parallel-channel model (see the Supplementary Materials for model calculations and explanation), and actual response probabilities (i.e., the proxy for detected visual contrast) of fiddler crabs to looming stimuli based on (**C**) varying intensity contrasts, (**D**) varying polarization contrasts, and (**E** and **F**) mixed intensity and polarization contrasts. The ranges of contrasts presented in (E) and (F) are the same as in (C) and (D) but with the addition of a fixed polarization or intensity contrast, respectively. Error bars are Wilson score intervals calculated using the sample size for each point (*n*) and the number of responses. Vertical dashed line is the location of zero contrast between stimulus intensity [for (C) and (E)] or stimulus polarization [for (D) and (F)] and the background. The data from two separate experiments are presented in (E), each with a different range of Weber contrasts. Note that the magnitude of response to any given stimulus depended on its contrast relative to that of the other stimuli tested within the same experiment rather than its absolute contrast. This is illustrated by comparing the response to the stimuli colored blue in (D) and (E), both of which have exactly the same polarization contrast (intensity contrast is zero). *n* is the number of animals that contributed to the response probability measured for each contrast.

If polarization and intensity information are processed within either a single channel or parallel channels, then several predictions can be made about the probability of an individual responding to a controlled stimulus that comprises both intensity and polarization. If both forms of information are combined into a single measure of contrast, then the addition of a fixed polarization contrast to a range of intensity contrasts (or vice versa) would cause a shift in the response minimum (Fig. 3A; see the Supplementary Materials for model calculations and explanation). Rather than falling to a minimum at the zero-contrast location on the *x* axis, the curve would be shifted to the left or right (depending on the polarity of the combination), revealing the contrast point where intensity and polarization cancel each other out. Alternatively, if polarization and intensity are processed in discrete and parallel channels, then the model would predict an upward shift in the response minimum (Fig. 3B), as such a system would not suffer from cancellation points.

To test which of these models of visual processing functions in fiddler crabs, a spherical treadmill (*21*, *22*) was used to investigate the response of fiddler crabs to visual stimuli differing in intensity contrast and/or polarization contrast. Each stimulus consisted of a looming circle that simulated the approach of a predator. The polarization and intensity properties of these stimuli could be adjusted independently using a novel type of display. Briefly, a patterned vertical alignment type liquid crystal display (PVA-LCD) was modified by removing the front polarizer to control the degree of polarization of transmitted light (the angle of polarization was always horizontal). This was spatially and temporally synchronized with a superimposed image from a digital projector that provided an intensity-based illumination source to produce a single image. In accordance with previous studies on fiddler crabs, we used behavioral response probability as a proxy for the visual contrast detected by the animal (*21*, *22*). Differences in intensity between the stimulus and the background are reported as Weber contrasts. Polarization contrasts were calculated using an opponent processing model, in which the horizontal and vertical channels act as excitatory and inhibitory units, respectively, producing a value of polarization distance for each stimulus/background combination (*7*).

Crabs responded strongly to both intensity-only and polarization-only looming stimuli, and the response probability was positively correlated with the magnitude of the Weber contrast [likelihood ratio test (LRT), χ^2^_(1)_ = 55.5, *P* < 0.001; Fig. 3C] and polarization distance [LRT, χ^2^_(1)_ = 19.12, *P* < 0.001; Fig. 3D], respectively. In both cases, crabs responded to contrasts asymmetrically, with a greater response probability to negative Weber contrasts (i.e., when the stimulus was darker than the background) than to positive, and to negative polarization distances (i.e., less polarized than the horizontally polarized background) than to positive. The shapes of the intensity-only and polarization-only response curves were similar (compare Fig. 3, C and D).

To determine which shift in response probability occurs, we repeated the experiments with the addition of a fixed polarization or intensity contrast, respectively (I + fixed P and P + fixed I). In both cases (Fig. 3, E and F), the results showed an upward shift in the response probability, and there was no evidence of any cancellation points. This is supported by the fact that there was no longer a significant effect of Weber contrast [I + fixed P: LRT, χ^2^_(1)_ = 0.39, *P* = 0.533; Fig. 3E, black dots]. Similarly, the effect of polarization contrast was also reduced [P + fixed I: LRT, χ^2^_(1)_ = 5.38, *P* = 0.02; Fig. 3E]. To confirm that a cancellation point had not been missed due to coarse sampling along the intensity contrast scale, we repeated the I + fixed P experiment using a narrower intensity range with the same result [LRT, χ^2^_(1)_ = 0, *P* = 0.997; Fig. 3E, gray squares].

**Fig. 4:**
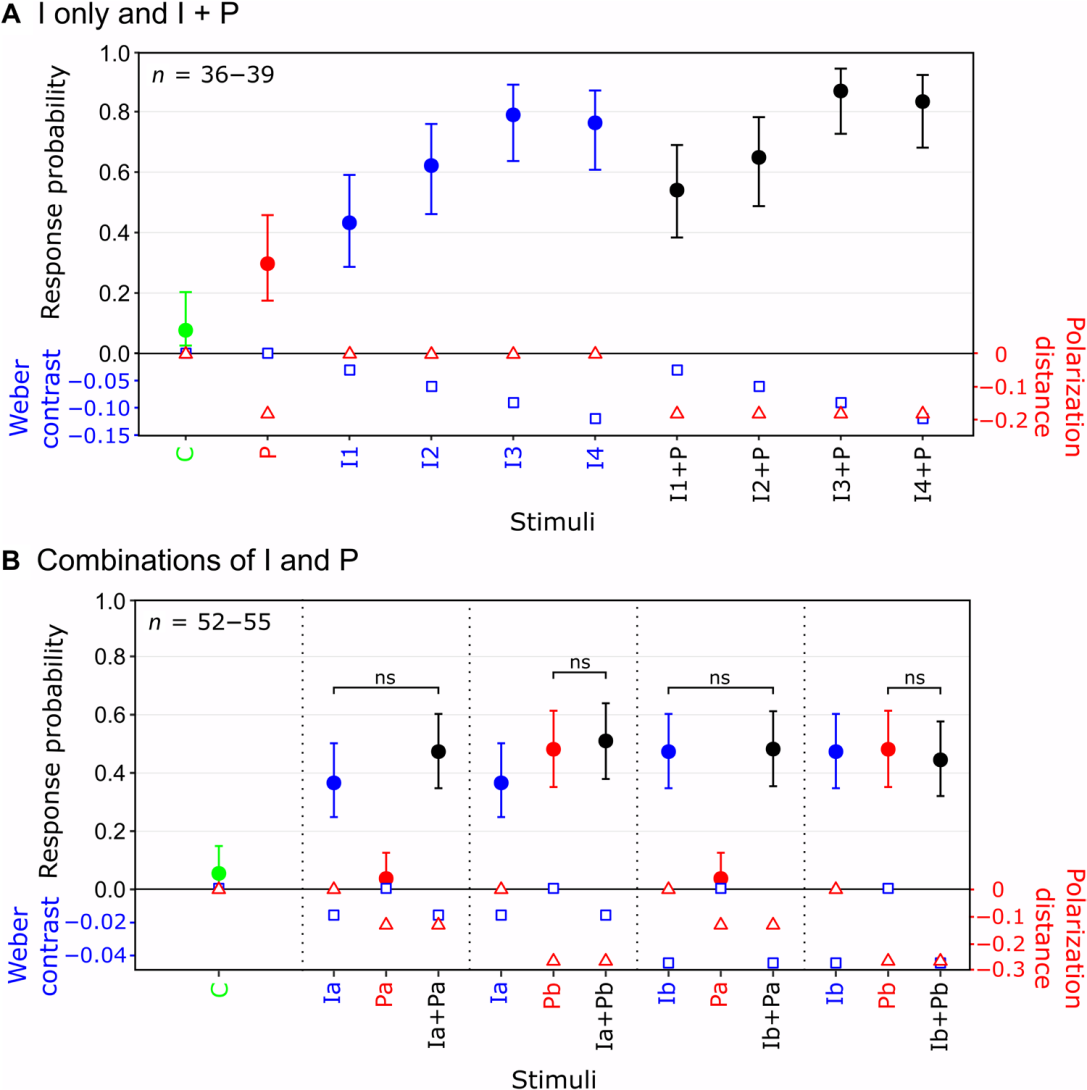
Interactions between intensity and polarization contrasts. (**A**) Addition of a fixed polarization contrast (P) to a range of intensity only stimuli (I1 to I4). (**B**) Solo and combined effect of two intensity contrasts (Ia and Ib) and two polarization contrasts (Pa and Pb). Note that in (B), for clarity, each of the I- and P-only stimuli is plotted twice, once for each stimulus combination. C, control (no intensity or polarization contrast). Error bars are Wilson score intervals calculated using the sample size for each point (*n*) and the number of responses. Nonsignificance (ns) between the highest solo response probability and combined probability was determined using pairwise McNemar tests. Intensity and polarization contrast levels for each stimulus are plotted on the lower-most axes (blue squares, Weber contrasts; red triangles, polarization distance). *n* is the number of animals that contributed to the response probability measured for each contrast.

Note that the response probability of a crab to any given stimulus depended on its contrast relative to that of the other stimuli tested within the same experiment rather than its absolute contrast, thus making it difficult to directly compare the magnitude of response probability between experiments (e.g., the contrast of the stimuli colored blue in Fig. 3, D and E, is exactly the same but produces a different probability of response). Therefore, to probe the interaction between the intensity and polarization channels further, we presented multiple combinations of intensity and polarization contrasts to crabs within single experiments. When a near-threshold polarization contrast (P) was added to a series of intensity contrasts (I1 to I4), it did not significantly boost response probability [LRT, χ^2^_(1)_ = 1.97, *P* = 0.161 when data from the control (C; no intensity or polarization contrast) and P only were excluded from the model; Fig. 4A]. This is consistent with the results in Fig. 3, C and E, that show little or no change in response to the four darkest intensity stimuli (i.e., those with a negative Weber contrast) following the addition of the polarization contrast. Furthermore, responses to combinations of two near-threshold intensity (Ia and Ib) and polarization (Pa and Pb) contrasts showed that, rather than interacting in an additive or multiplicative fashion to affect response probability, combined stimuli were no more effective at eliciting responses than the most contrasting channel on its own (Fig. 4B).

## DISCUSSION

When detecting a moving object, the fiddler crab *A. tangeri* processes polarization and intensity contrast separately and in parallel. It does not process these two visual dimensions as a single form of contrast, as previously hypothesized for other crustaceans (*16*, *17*). The key advantage of this method of processing polarization and intensity in a parallel system is that the separate channels of intensity and polarization provide a greater range of detectable contrast information for the receiver. Previous work has shown that crabs use polarization information for target detection within their natural habitat (*2*), and processing polarization and intensity in parallel channels would enhance the detection of a moving target by providing two alternative, nonconflicting, sources of information, overall increasing the chance of the crab spotting a potential threat. Such a parallel processing architecture does not suffer from the cancellation points inherent in the single-channel model, allowing the receiver to benefit from the more consistent polarization information (see Fig. 2), without it interfering with the perception of intensity. Meanwhile, the separate intensity channel will be particularly important when polarization information is not available, for instance, when detecting a bird against a cloudy sky.

In the context of the animal’s sensory ecology, the crab’s initial anti-predator freeze response (which was used in this study as a proxy for the visual contrast detected) depends on whatever contrast is the most salient and above a certain response threshold, whether it be intensity or polarization. For instance, the addition of the fixed polarization contrast in Fig. 3E only increased the response to the stimuli with the lowest Weber contrasts, indicating that, in these cases, it was the polarization contrast that was most salient, while at higher Weber values the intensity contrast remained the most salient cue, and so the addition of polarization appeared to have little effect. This is further supported by the result of the second set of experiments (Fig. 4), which show that when intensity and polarization contrasts were combined, the resulting response probability was the same as that to the most contrasting solo contrast, regardless of whether it was in intensity or polarization.

An additional finding of interest is the similar asymmetry in the probability of response for the intensity-only (Fig. 3C) and polarization-only (Fig. 3D) experiments. The crabs were always more responsive to looms with a negative contrast (dark on light and less polarized on more polarized). This asymmetric response to intensity contrasts has been well documented in other species from various taxa (*23*–*26*). The implication of this is that if a crab were approached by a bird with a weak positive intensity contrast, then the polarization contrast would still be negative (Fig. 2) and so likely be the most salient cue in this instance; this further strengthens the argument that, when present, polarization can be a more reliable channel for detecting predators than intensity. The reliability of polarization information for target detection may also be an important driver behind the evolution of polarization vision in species from other taxa such as *Papilio* butterflies (*27*).

**Fig. 5:**
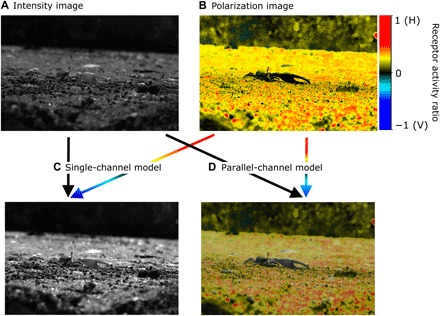
Image processing inspired by single- and parallel-channel models. Intensity (**A**) and (**B**) polarization images are combined, (**C**) to enhance intensity contrast through the single-channel model or (**D**) as separate layers of contrast information using the parallel-channel model. Photo credit: Martin How, University of Bristol.

If we consider the neural substrate, then what evidence is there to support the parallel-channel model? The photoreceptor projections from the R1 to R7 terminate in the lamina where they synapse with monopolar cells within the external plexiform (epl1 and epl2). The layer in which each set of receptors terminate may differ between taxa [compare (*14*, *28*, *29*) with (*30*, *31*)]. In the crayfish *Procambarus clarkia*, and gonodactyloid stomatopods, the horizontal receptors (R1, R4, and R5) terminate in epl1 and have synaptic sites with monopolar cell 3 (M3; Fig. 1A, red), while the vertical receptors (R2, R3, R6, and R7) terminate in epl2 and have synaptic sites with M4 (Fig. 1A, blue) (*30*, *31*). Together, opponent-processed outputs from M3 and M4 would form an intensity-independent polarization channel. M2 has postsynaptic sites across both epl1 and epl2 with all seven photoreceptors (Fig. 1A, black) and is likely responsible for summing the inputs from both photoreceptor orientations to form a polarization-independent intensity channel (*14*, *15*). M2, M3, and M4 all terminate in the medulla (*14*, *15*), at which point how the information is processed becomes less clear. In order for polarization contrasts to be determined, a mechanism of polarization opponency between the orthogonally orientated photoreceptors is first required (*7*). This opponent mechanism almost certainly occurs within the crab’s medulla, where polarization opponent neurons (POL-neurons) likely receive antagonistic input from M3 and M4. The existence of POL-neurons has been studied in the medulla of crickets and locusts (*32*–*34*), and while comparably less is known about POL-neurons in crustaceans, polarization-sensitive interneurons have been identified in the medulla of the crab *Scylla serrata* (*35*), and tangential cells in the medulla of crayfish have been shown to exhibit polarization opponency (*36*, *37*). Following this initial opponency between orthogonally orientated photoreceptors, a measure of polarization contrast between different ommatidia (e.g., one viewing the object and the other the background) can be determined. Speculation about the neural substrate involved with processing the separate intensity and polarization contrasts past this point is beyond the scope of this study. However, these results do suggest that the freeze response displayed by fiddler crabs during the first stage of their anti-predator response is likely controlled by a biphasic OR gate that receives two inputs: one from neurons relaying information on intensity contrast and the other information on polarization contrast. The OR gate would fire when one or both of these inputs are above a specific threshold. This parallel method of visual processing enables fiddler crabs to benefit from the advantages of both intensity and polarization information while simultaneously mitigating the weaknesses of both. Furthermore, although not directly comparable, the separate channels for polarization and intensity may be thought of as being analogous to the well-studied M (magnocellular) and P (parvocellular) pathways of humans and Old World monkeys that are generally considered to function as separate channels for intensity and color information, respectively (*18*). Like the M and P pathways, the polarization and intensity channels start separately but likely converge at a later stage of visual processing to enable behavioral decision making. This therefore raises the possibility that they may be combined to form an image with separate layers of contrast information (Fig. 5) analogous to how humans and other animals perceive intensity and color information. In addition, there is limited evidence to suggest that fiddler crabs are dichromatic (*38*), presumably via an opponency between the short-wavelength sensitive R8 receptor (which is not polarization sensitive) and the R1 to R7 receptors that are most sensitive to medium wavelengths (*39*). If this is the case, then future work might investigate whether intensity and polarization are integrated with potential color channels.

In summary, intensity and polarization information within a visual scene are processed independently in parallel channels. Each form of visual information therefore contributes its own measure of visual contrast, which then feeds into processing circuits that mediate visually guided behavior. This finding proves that crabs, perhaps along with other crustaceans, do not simply perceive polarization only as a modulation of the intensity information. Therefore, how these animals actually see polarization in terms of image forming is more complex and exciting than previously thought.

## METHODS

Fiddler crabs (*A. tangeri*) (carapace width, between 20 and 45 mm) were collected by hand from the mudflats of El Rompido, southwest Spain (37.2207°N, 7.1238°W), and housed separately in plastic cups with a small volume of seawater (changed daily). Crabs were kept for a maximum of 5 days under natural shade conditions and were fed with fish flake food once a day. A total of 285 crabs were tested across the whole study, and each individual crab was tested only once before being released at the site of collection.

**Fig. 6:**
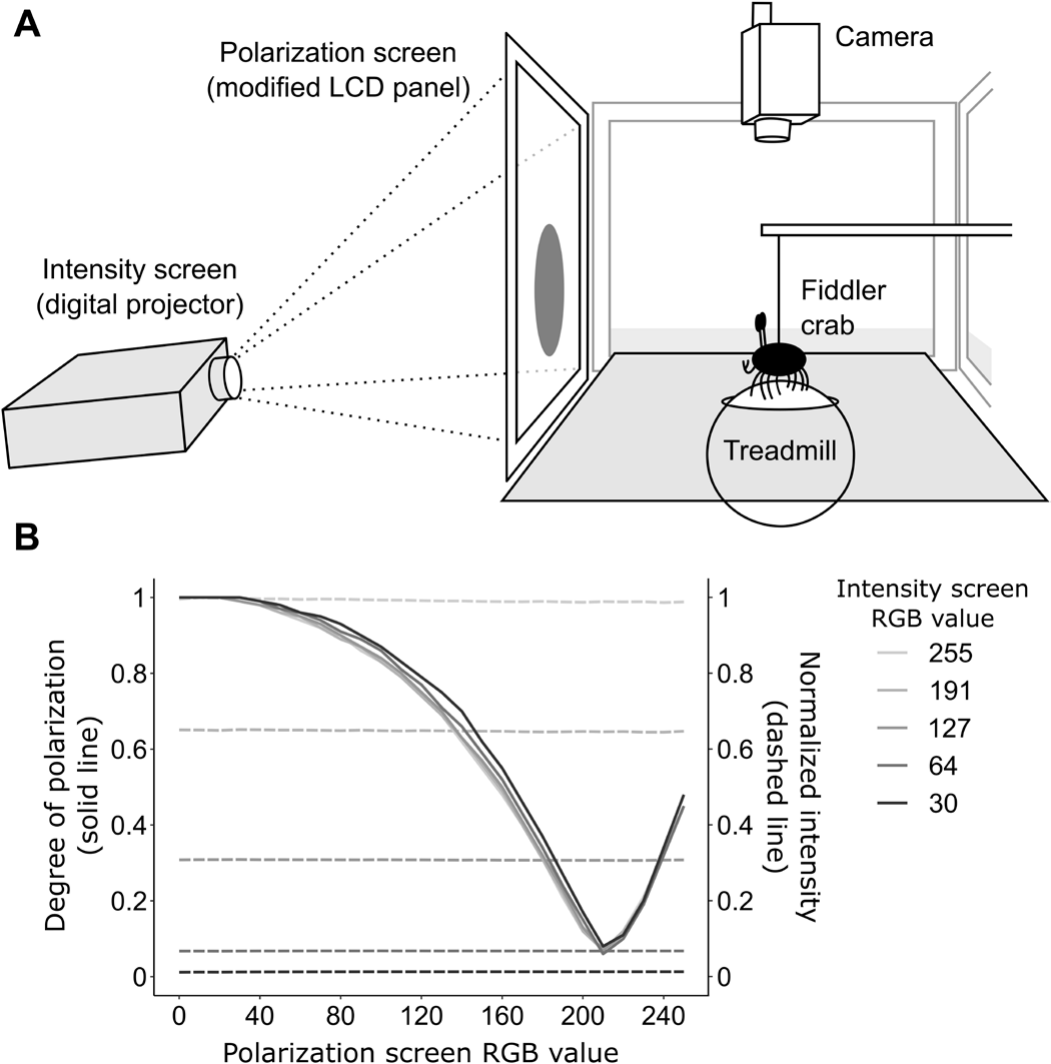
Experimental setup and properties of the IP screen. (**A**) Treadmill apparatus and IP screen. Crabs were subjected to looming stimuli that varied independently in intensity (produced by the digital projector) and polarization (produced by the modified LCD panel). (**B**) Polarization (solid lines) and intensity (dashed lines) measurements of the IP screen at different intensity and polarization screen RGB values (R = G = B).

Each crab was loosely tethered on top of a 150-mm-diameter Styrofoam treadmill (Fig. 6A) suspended on a cushion of air supplied by a nonheating hair dryer (BaByliss 3Q). This allowed the crabs to walk freely while preventing translational or rotational movement. Stimuli were presented to crabs using a custom-built intensity-polarization (IP) screen that allowed intensity and polarization contrasts to be adjusted independently. The screen consisted of two displays that were spatially and temporally synchronized: (i) a digital projector (CP-WX3030WN, Hitachi Ltd., Tokyo, Japan) that cast an intensity-based image onto a sheet of diffuser (#250 Half White Diffusion, Lee Filter, Andover, UK) on the rear surface of (ii) a modified patterned vertical alignment type LCD panel dissembled from its outer casing (1905FP, Dell, Round Rock, USA) and with the outermost polarizer removed (*40*). The IP screen thus allowed the degree of polarization and the intensity of an image to be controlled simultaneously and independently (Fig. 6B). For all experiments, the background was set to a degree of polarization of 0.5. The angle of polarization of both the background and the stimuli was always approximately horizontal (fig. S1). The IP screen was positioned directly in front of the crab, at a distance of 220 mm, and three other monitors (Dell 1905FP), two either side and one behind the crab, displayed a simulated visual horizon. A green filter with peak transmission at approximately 515 nm (#124, Lee Filters, Andover, UK) was positioned between the light source and the LCD panel of all the screens so that the output roughly corresponded with the peak visual sensitivity of the R1 to R7 photoreceptors of the crabs (λ max = 530 nm) (*39*). A calibrated spectrometer (QE65000, Ocean Optics, Largo, USA) was used to measure the irradiance values of the IP screen. These were then integrated and used to calculate the Weber contrast of the stimulus/background combinations. Polarization properties were measured using a rotatable Glan Thompson polarizer coupled to the spectrometer. Irradiance levels were measured through the polarizer at angles of 0°, 45°, 90°, and 135° and combined to calculate the polarization distance (*7*) between the stimulus and the background. The calculation of Weber contrast and polarization distance was based on the spectral sensitivity of the R1 to R7 photoreceptors for *A. tangeri* (*39*, *41*).

Looming stimuli, consisting of expanding discs above the crab’s visual horizon (to simulate an approaching predator), were presented to fiddler crabs using a fully automated protocol developed in MATLAB (R2015a and R2016a, MathWorks, Natick, USA). After a 2-min acclimation period on the treadmill, each crab was presented with 9 or 10 stimuli (depending on the experiment) in a fully randomized order, with minimum between-stimulus intervals of 20 s plus a random pause of up to 20 s (any effect of habituation was controlled for in the statistical analysis and by the randomization of the stimulus order). This pause was longer if the crab was stationary, as MATLAB was programmed to check that the crab was walking before initiating the next presentation (see description of motion detection system below). The looming stimulus expanded exponentially from a visual angle of 0° to 20° in a time of ~12 s. Behavior and treadmill movement were recorded from above using a webcam (C270, Logitech, Lausanne, Switzerland). Fiddler crabs show a multistaged escape response when approached by a potential threat (*42*, *43*); the crab’s initial freeze response was used as a proxy for perceived visual contrast (see movie S1). Response was scored automatically in MATLAB at the end of each presentation using a two-dimensional motion detection algorithm (*44*), which detected the motion of markings drawn on the polystyrene ball. The crab’s response was scored within a 4-s window, 2 s before max loom size and 2 s after. As the crab’s normal behavior on the treadmill was to maintain a steady walk, a response to the stimulus was recorded if the animal stopped walking during the scoring window. Trials in which the crab was not walking at stimulus onset were rejected, and the stimulus was appended to the end of the series for a repeat presentation (up to a maximum of five extra stimuli). Any effect of habituation was controlled for in the statistical analysis. Any remaining trials in which the crab stopped before the scoring window were rejected post hoc. To limit the amount of time each crab spent on the treadmill, and thus any associated stress or motor fatigue, the trial was ended after 30 min.

### Statistical analysis

A mixed-effects binary logistic regression was used to analyze the data from each experiment. The response variable was whether or not the crab responded. In specifying the maximum model, for the first set of experiments, either Weber contrast (Fig. 3, C and E) or polarization distance (Fig. 3, D and F) was included as a continuous fixed effect. In the second set of experiments (Fig. 4), both Weber contrast and polarization distance were included. Crab sex, size, and the presentation number (order) were included as additional fixed effects. The latter was included to control for any effect of habituation. Crab identification (to account for repeated measures) was included as a random effect. We used model simplification to test for significant fixed effects, whereby models were compared with one another using an LRT to sequentially remove nonsignificant effects. While some of the experiments did show evidence of habituation, I + fixed P [Fig. 3E, black dots: χ^2^_(1)_ = 12.71, *P* < 0.001; gray squares: χ^2^_(1)_ = 7.04, *P* = 0.008], P + fixed I [Fig. 3F: χ^2^_(1)_ = 4.12, *P* = 0.043], and the combinations of I and P [Fig. 4B: χ^2^_(1)_ = 7.45, *P* = 0.006], both the analyses controlled for this and the randomization of stimulus order means that the overall effect of habituation on response probability would have been the same for all stimuli. There was no effect of size or sex except for a very weakly significant effect of sex for I + fixed P [Fig. 3E, black dots: χ^2^_(1)_ = 4.05, *P* = 0.044].

Last, for the combinations of I and P experiment (Fig. 4B), pairwise McNemar tests were used to assess whether combined stimuli were more effective at eliciting responses than the most effective solo contrast.

## Supplementary Material

http://advances.sciencemag.org/cgi/content/full/5/8/eaax3572/DC1

Download PDF

Movie S1

Data file S1

MATLAB code

## SUPPLEMENTARY MATERIALS

Supplementary material for this article is available at http://advances.sciencemag.org/cgi/content/full/5/8/eaax3572/DC1 Fig. S1. Angle of polarization (AoP) of the IP screen. Fig. S2. Top-view schematic of the two-channel polarization camera used to capture video of seabirds. Fig. S3. Simulation results from the IP response model showing the normally disputed response thresholds. Fig. S4. Example predictions from the IP response models. Movie S1. Example freeze response of a fiddler crab to a looming stimulus. Data file S1. Data from behavioral experiments. MATLAB code for running the IP response model Reference (*47*)
