# Supplementary material for: Parallel processing of polarization and intensity information in fiddler crab vision
Source: Sci Adv. 2019 Aug 21;5(8):eaax3572. doi: 10.1126/sciadv.aax3572 (PMC6703871; doi:10.1126/sciadv.aax3572)
Supplement: http://advances.sciencemag.org/cgi/content/full/5/8/eaax3572/DC1 [file supp_5_8_eaax3572__index.html]

Science Advances | Science AdvancesAAASSearchScience AdvancesMenu

## Supplementary Materials

**The PDF file includes:**

- Fig. S1. Angle of polarization (AoP) of the IP screen
- Fig. S2. Top-view schematic of the two-channel polarization camera used to capture video of seabirds.
- Fig. S3. Simulation results from the IP response model showing the normally disputed response thresholds.
- Fig. S4. Example predictions from the IP response models.
- Legend for movie S1
- Legend for data file S1
- Legend for matlab code
- Reference (*47*)

Download PDF

**Other Supplementary Material for this manuscript includes the following:**

- Movie S1 (.avi). Example freeze response of a fiddler crab to a looming stimulus.
- Data file S1 (Microsoft Excel format). Data from behavioral experiments.
- MATLAB code for running the IP response model (.m format).

**Files in this Data Supplement:**

- Adobe PDF - aax3572\_SM.pdf
