# Supplementary material for: Parallel processing of polarization and intensity information in fiddler crab vision
Source: Sci Adv. 2019 Aug 21;5(8):eaax3572. doi: 10.1126/sciadv.aax3572 (PMC6703871; doi:10.1126/sciadv.aax3572)
Supplement: Download PDF [file aax3572_SM.pdf]

Science Advances
AAAS

advances.sciencemag.org/cgi/content/full/5/8/eaax3572/DC1

Supplementary Materials for

Parallel processing of polarization and intensity information in fiddler crab vision

Samuel P. Smithers*, Nicholas W. Roberts, Martin J. How*

*Corresponding author. Email: sam.smithers@bristol.ac.uk (S.P.S.); m.how@bristol.ac.uk (M.J.H.)

Published 21 August 2019, *Sci. Adv.* **5**, eaax3572 (2019)
DOI: 10.1126/sciadv.aax3572

The PDF file includes:

Fig. S1. Angle of polarization (AoP) of the IP screen.
Fig. S2. Top-view schematic of the two-channel polarization camera used to capture video of seabirds.
Fig. S3. Simulation results from the IP response model showing the normally disputed response thresholds.
Fig. S4. Example predictions from the IP response models.
Legend for movie S1
Legend for data file S1
Legend for matlab code
Reference (*47*)

Other Supplementary Material for this manuscript includes the following:

(available at advances.sciencemag.org/cgi/content/full/5/8/eaax3572/DC1)

Movie S1 (.avi). Example freeze response of a fiddler crab to a looming stimulus.
Data file S1 (Microsoft Excel format). Data from behavioral experiments.
MATLAB code for running the IP response model (.m format).

Angle of polarization of the intensity-polarization screen

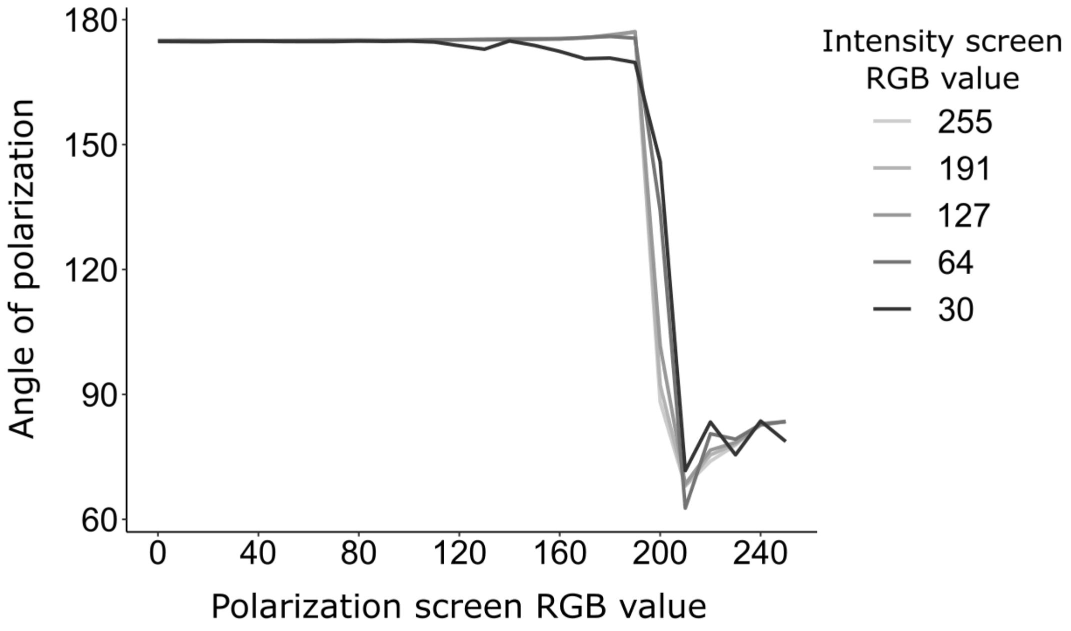

Fig. S1. Angle of polarization (AoP) of the IP screen. An angle of 0°/180° indicates the light is horizontally polarized and an angle of 90° indicates it is vertically polarized. Below an RGB value of ~180 on the polarization screen the AoP is approximately horizontal however above this greyscale value the AoP flips to near vertical. For this reason the RGB values of the polarization screen used in this study were well below 180.

Polarization camera

Polarization video data was collected using a custom-built camera system, comprising two usb-controlled cameras (UI-3240CP-NIR-GL, IDS, Obersulm, Germany), a polarization beam-splitter (CCM1-PBS25/M, Thorlabs, Newton, USA) and a lens (Arsat 1:35, 30mm, Kiev Cameras, Ukraine). The two cameras were temporally synchronised using a custom-made master/slave hardware-trigger system and manually controlled exposure settings. All other automatic image processing systems (e.g. gain) were disabled. The two cameras were mounted directly onto two faces of the polarization beam-splitter cube so that one camera received the horizontally polarized component of the visual scene and the other, the vertical (fig. S2). A wide-angle lens was mounted on the third surface of the beamsplitter cube to counter the reduced field of view caused by a) lengthening the focal distance from the lens to the camera CCD chip, and b) the small dimensions of the camera chip. Horizontal and vertical video frames were then processed using custom-written software (Matlab 2018b, Mathworks, Natick, USA). Images were first registered using a feature-detection technique (function *detectSURFFeatures* from the vision toolbox). Each pixel in horizontal/vertical video frame pairs were then used to calculate a measure of receptor contrast, based on the formulae of Bernard and Wehner (1977) (*6*) and How and Marshall (2014) (*7*). False colours were then assigned to the range of possible values of receptor contrast to illustrate the contrast available to the fiddler crab visual system. It should be noted that caution should be taken when calculating the polarization properties of dark objects or areas of a scene because instrument noise in the measurements can result in significant artefacts and incorrect conclusions about high degrees of polarization when in fact none exist (*47*).

The image in Fig. 2 was taken at approx. 13:10 on the 2nd Nov 2018 at Western-Super-Mare, UK (51.34212900,-2.98248600). The elevation and azimuth of the sun were 24° and 184° respectively. Because the camera was not kept stationary during filming (but

instead followed the birds across the sky) we do not have accurate data on the filming direction and elevation of the camera at the point the image in Fig. 2 was taken.

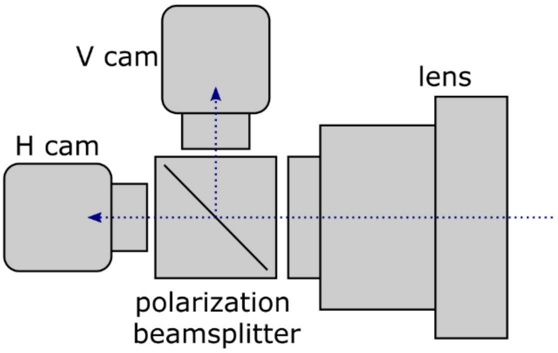

Fig. S2. Top-view schematic of the two-channel polarization camera used to capture video of seabirds. H and V cam are USB-controlled cameras detecting horizontal and vertical components of the visual scene filtered by the polarization beamsplitter cube. Blue dotted arrows indicate the path of light through the lens, beamsplitter and camera.

Intensity-Polarization (IP) response model

Here we outline a simple model for predicting the response of crabs to combinations of intensity and polarization contrasts (full Matlab script can be downloaded from the supplementary materials). The model simulates a population of 10,000 virtual crabs, assigns them a threshold level of response to positive and negative contrasts in intensity and polarization (based on the experimental observations in this study), then estimates the probability of response to combinations of intensity and polarization using either a single channel or a parallel channel integration system. The following steps illustrate how the model is formulated.

Step 1 – Simulate a population of crabs and assign each with a threshold for responding to positive and negative intensity and polarization contrasts. 10,000 normally distributed

threshold values were generated for four different situations: negative and positive weber contrasts of -0.0548 (± SD 0.0204) and 0.162 (± 0.0417) and polarization distances of -0.333 (± 0.111) and 0.846 (± 0.374) to approximate the response curves measured in the behavioural experiments (Fig. 3c-d). These threshold distributions were derived by fitting sigmoidal curves (using the 'sigm_fit' function in Matlab https://uk.mathworks.com/matlabcentral/fileexchange/42641-sigm_fit) to the negative and positive results in the intensity-only and polarization-only behavioural experiments (Fig. 3c-d). The centre for each threshold distribution was derived from the mid-point of each fitted sigmoidal curve, and the standard deviation was scaled relative to sigmoidal slope using the relationship,

$$SD = 0.4/slope$$

in order to approximate the slopes observed in the behavioural data. Threshold distributions were generated using the 'normrnd' function in Matlab, which returns an array of random numbers following a normal distribution with a specified peak location and standard deviation. The distribution of response thresholds for both intensity and polarization contrasts is illustrated in fig. S3a-b (red shaded areas for negative and green for positive contrasts). In addition to this, type 1 errors (crab responds to a sub-threshold stimulus) and type 2 errors (no response despite an above-threshold stimulus) were simulated by switching the response of 5% of all simulated trials, selected randomly using the 'rand' function in Matlab. This results in a response probability curve across the simulated population which approximates that observed in the experimental data (fig S3 a-b, black lines).

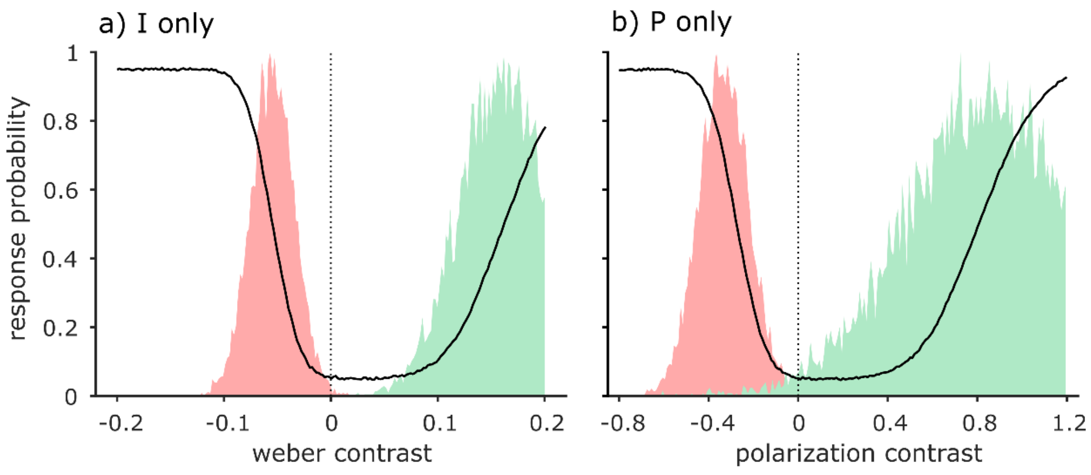

Fig. S3. Simulation results from the IP response model showing the normally disputed response thresholds. Illustration of the IP response model in intensity-only (a) and polarization-only (b) simulations. Normally distributed response thresholds (red (negative) and green (positive) shaded areas – normalised to y-axis range) approximating those observed in the behavioural experiments were used to simulate the response profile of a population of 10,000 crabs. A random sub-sample of 5% of simulated trials had their response switched to simulate a small chance of type 1 and type 2 error. This resulted in a population-level response curve (black line) that increases either side of the zero contrast value (dotted line).

Step 2 – Combine intensity and polarization contrasts using the single channel and parallel channel models. Combinations of intensity and polarization are processed using one of two methods:

a) *Single channel model*: first the polarization contrast measure is modified by a conversion factor to bring the polarization distance scale approximately equivalent to the weber contrast scale. A conversion factor of 0.17 is used for this example, a value derived by using a least sum-of-squares approach to compare the behavioural response probabilities in Fig. 3c and 3d. The intensity and modified polarization contrasts are then summed and crab response

rescored according to the intensity-only thresholds outlined in fig. S3a. The addition of a fixed polarization contrast to a series of intensity contrasts would then result in a sideways shift of the response curve, the direction of which depends on the polarity of the I-P combination (fig. S4a and Fig. 3a).

b) *Parallel channel model*: in this case, if either intensity or polarization contrasts are above threshold, then the animal responds. If both are below threshold, then no response is observed. This results in an upwards shift of the response curve (fig. S4b and Fig. 3b).

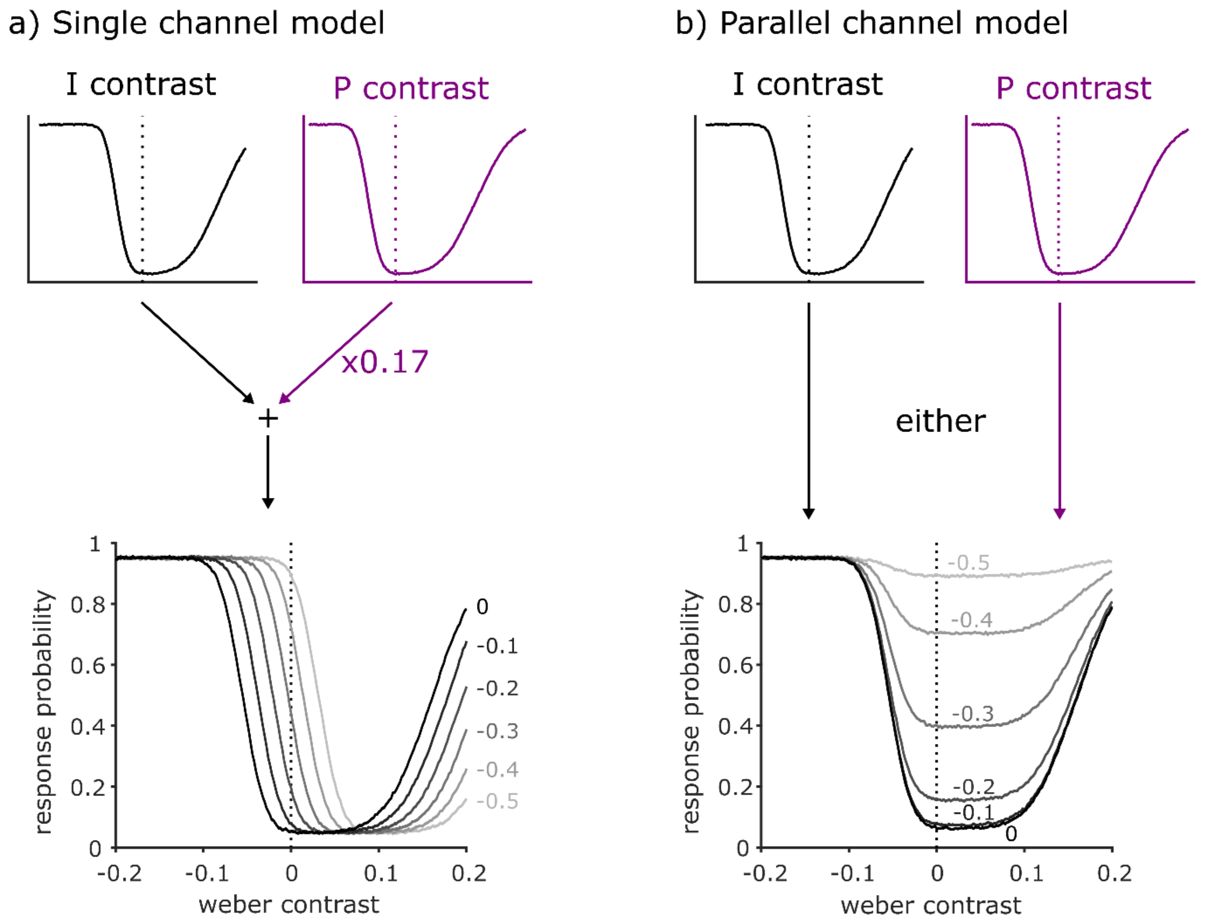

Fig. S4. Example predictions from the IP response models. Combination of intensity and polarization contrasts using the single (a) and the parallel (b) channel models. For the single channel model (a), I and P contrasts are added together (after scaling the polarization distance

measure by a factor of 0.17) to form a single measure of contrast. For the parallel channel model (b), each channel contributes a separate estimate of contrast and responses are elicited if either are above threshold. Bottom two graphs illustrate the effect on response probability of adding five different fixed polarization contrasts (grey lines and values) to a series of intensity contrasts.

Additional supplementary materials

Movie S1. Example freeze response of a fiddler crab to a looming stimulus.

Data file S1. Data from behavioral experiments.

MATLAB code for running the IP response model. (file name: "IP_fiddlerIPmodel3_methodsfig.m").
